# Selection of suppliers using crisp gradual means integral in conjunction with clustering algorithms

**DOI:** 10.1016/j.mex.2023.102442

**Published:** 2023-10-14

**Authors:** Abdelhakim Abdelhadi, Mohammed Almomani

**Affiliations:** a Prince Sultan University, Rafha St., Riyadh, Kingdom of Saudi Arabia; b Jordan University of Science and Technology, Irbid, Jordan

**Keywords:** Clustering algorithms, Supply chain management, Similarity coefficient, Group technology, Fuzzy analytic hierarchy processes, Crisp Gradual Means Integral in Conjunction with Clustering Algorithms

## Abstract

It is important for companies to select the right suppliers to deal with in order to avoid any disruptions. Considerable research has been conducted to find the solution for this problem. Previous attempts were made to account for uncertainties and disruptions in decision-making in order to rank suppliers based on different criteria of interest and select the desired resources as needed. This study addresses this issue by grouping suppliers in groups based on their similarities in terms of decision-making standards (criteria). Crisp Gradual Means Integral used in fuzzy analytic hierarchy process is used in conjunction with the clustering technique. By grouping suppliers into clusters, several advantages can be gained by the decisions’ maker firms. For example, a firm can determine the level of similarity between different suppliers when taking into consideration all variables of interest to its decision-makers. When computing this similarity, the decision-makers can substitute any of the suppliers with other supplier from the same group in the event of the occurrence of a negative event that prevents the original selected supplier from fulfilling their obligations.

• The crisp gradual means integral is a well know approach used in the fuzzy analytics hierarchy processes.
• The proposed methodology adapts the crisp means integral and clustering algorithms.
• The resulted outcomes group suppliers into groups to help the decision makers select the best supplier.

## Graphical abstract

**Figure:**
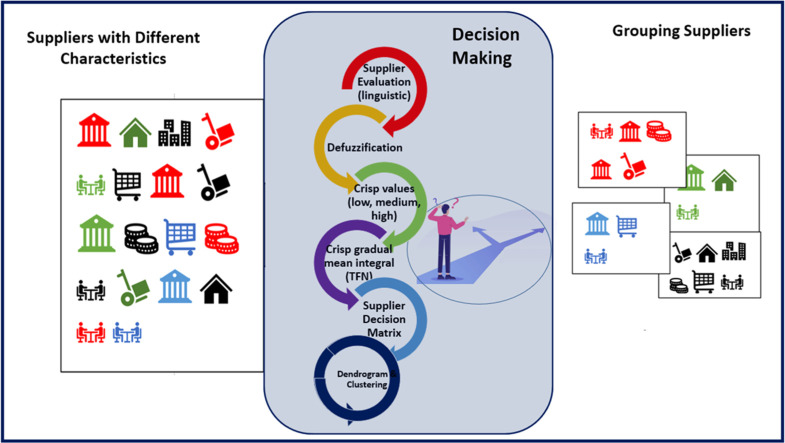

Specifications table

**Table:** 

Subject area:	Engineering
More specific subject area:	*Supply chain management*
Name of your method:	Crisp Gradual Means Integral in Conjunction with Clustering Algorithms
Name and reference of original method:	*NA*
Resource availability:	*NA*

**Method details**

## Introduction

Coordination and exchange of information among supply chain partners increase the complexity of opportunities that lead to increased cost and risk in the supply of required materials [[1], [2]] which may result in a loss of flexibility in the arrival of goods and supplies [3]. Recently, the effects of the COVID 19 epidemic crisis and global geopolitical tensions, such as the Ukrainian and the Russian war have caused the phenomenon of supply chain disruption by forcing governments and industries to adopt forms of protectionism which led to a slowdown in international economic trade after decades of increase integration [4]. Alvstam and Fang, [5] stated that manufacturing enterprises should maximize the use of production components and reconfigurea supply chains using resupply and approximation strategies in this global crisis environment and disruptions in natural and energy supply chains resources. The system of creating and capturing value has fundamentally changed, it requires significant effort by industries to reinvent organizational processes and models, products, and business models [6] in a sustainable perspective [7] to respond to the unexpected market shock, therefore it is necessary to diversify sources of supply and create new supply chains more flexible, agile [[8], [9]] and green [10], as well as low carbon supply chain footprint [[11], [12]], for example, Miguel et al. [4] showed how bringing extraction sources closer to factories significantly reduces CO2 emissions to the atmosphere from transportation. This research will contribute to minimizing the effect of the adverse situations due to unseen developments in the supply chain management, by introducing the concept of merging two methodologies together which will help the decision makers to have more flexibility in giving weights to the available alternatives.

Thus, in response to these risks, firms began to develop supply chain risk management approaches, for preventing this adverse situation and for responding more appropriately to supply chain risks; these approaches are used for improving the firms’ agility when dealing with the above issues [[13], [14]]. Decision-makers achieve positive coordination in the supply chain, act rationally, and make decisions that are effective for the entire supply chain system and the entire company range [15]. The goal of such coordination is to synchronise all activities to work uniformly and to improve the overall performance of the supply chain, by managing the interactions between the different entities involved in the system [16]. The choice of the supply coordination methodology is therefore important for ensuring successful performance of the entire supply chain and company-wide operations. A supply chain can be thought of as a decision-making process that leads to the success or failure of the business because of the above-mentioned risks; thus, failure to use a multi-criteria decision-making process could make it difficult for managers and decision-makers to choose a suitable method that suits their needs.

The survey of existing literature revealed that researchers and academics use different methods to prioritise suppliers and classify them according to the decision-makers' needs, such as distances or their effect on the environment. In this work, an attempt to join suppliers based on the highest similarities between them, based on the objectives of the decision-makers, which in turn eases the supply-chain processes and alleviates the above-mentioned problems. This paper attempts to explore priority coordination mechanisms and prioritisation of selected partners using group technology methods based on using Crisp Gradual Means Integral which is a method being used as conversion measures to measure the density of positions developed by decision-makers in the form of linguistic terms. Several researches were conducted in the field of using clustering to solve the supply chain management issue. Ryder and Fearne applied clustering methods in conjunction with supply chain management and presented a case study in the food industry [17]. Che proposes optimization mathematical models for the clustering and selection of suppliers based on performs an analysis of supplier clusters, according to customer demand attributes, including production cost, product quality and production time [18]. Park and Lee used a hybrid approach for evaluation and selection is presented using an expectation maximization (EM) algorithm for clustering, data envelopment analysis (DEA) for efficiency, and analytic hierarchy process (AHP) for importance [19]. Nicolas, et al. used interval type-2 fuzzy values to show the decision makers’ preferences [20]. Kara and Firat, study proposes a clustering-based approach to group suppliers based on their risk profile [21]. This research will response positively to the changes of the variability in the risks of the of each entity and deals with coordination of the supply chain process dynamically.

## Multi-criteria decision-making

Various decision-making approaches and methods have been widely used among academics to select suppliers, wherein all quantitative and qualitative variables are considered during the final selection of decision-makers [[22], [23]]. Currently, uncertainty and sustainability are one of the most important issues in the selection of suppliers. Ashkan et al. used the developed Firefly algorithm to alleviate the supply chain network problem, for meeting the needs of decision-makers [24].

Hamel and Valikangas [25] use the concept of flexibility, where the flexibility variable acted to restore the intermittent system to its original state or another, better than the original, state. A quantitative approach was used for selecting the best strategic resource for a mystery system by Van Laarhoven and Pedrycz [26], who suggested to use a fuzzy analytic process (FAHP), which is a combination of the mathematical analytical hierarchy process (AHP) and fuzzy theory.

Wang and Yang [27] have used multi-objective linear programming, involving and relying on the theory of fuzzy AHP, to select appropriate resources in a quantitative discount environment. Kilincci and Onal [28] modelled the choice of fuzzy AHPs for a washing machines company, based on many criteria and decision-making. Zadeh [29] proposed a fuzzy theory of positioning, where all of the information captures a specific subjective subject and is translated into numbers representing a set of certain values based on their importance, while decision-making trial and evaluation laboratory (DEMATEL) is used to obtain qualitative assessments from multiple experts regarding how to mitigate the impact of the barriers [30]. The proposed modelling using fuzzy clusters has proved to be an effective means for formulating decision problems related to the problem under study, where the available information is subjective and accurate. Fuzzy indices correspond to a certain range of a given value. The specific range for a decision-maker allows to assess his/her preferences easily based on the nature of the matter.

One linguistic translation and evaluation is translated into a fuzzy number consisting of multiple numbers, depending on the importance of the problem under consideration. In this way, the language classification is reflected as a range of numerical numbers. Both triangular and trapezoidal fuzzy numbers can be used in the fuzzy approach. Balli and Korukoglu [31] presented the advantages of using fuzzy numbers (TFNs) based on the ease of calculations. Chalekaee et al. [32] presented several MCDM methodologies when presented his research dealing with construction industries. Since basic AHP does not include vagueness for personal judgments, it has been improved by benefiting from fuzzy logic approach. In F-AHP, the pairwise comparisons of both criteria and the alternatives are performed through the linguistic variables, which are represented by triangular numbers [33]. One of the first fuzzy AHP applications was performed by van Laarhoven and Pedrycz [26]. They defined the triangular membership functions for the pairwise comparisons. Afterwards, Buckley [34] has contributed to the subject by determining the fuzzy priorities of comparison ratios having triangular membership functions. Chen [35] also introduced a new method related with the usage of triangular numbers in pairwise comparisons.

Although there are some more techniques embedded in F-AHP, within the scope of this study, Buckley‟s methods [34] is implemented to determine the relative importance weights for both the criteria and the alternatives.

## Grouping technology

The grouping approach is used in manufacturing; in this approach, common similarities between the characteristics of a group of things are identified and exploited, mainly the characteristics of parts under study, and these parts are collected in independent cells according to their characteristics [36]. Cellular manufacturing applies the grouping approach to manufacturing. In cellular manufacturing, machines are assembled based on their work in shaping the parts, which are usually differentiated in function, into cells. These cells must be ideally responsible for handling a set of parts called the family part. The idea is to treat a family of plug-in components through a dedicated cell in the ingenuity of those pieces, thus obtaining the advantages of mass production without the problem of mixing, which may lead to a significant waste of time. Yong Yin [37] examined and reviewed various similarity coefficients of the cell formation problem. The Jaccard similarity coefficient is the most commonly used common denominator. The Jaccard similarity coefficient [38] is defined between device *i* and device *j*, as follows:

(1) $$S_{ij}=\frac{a}{a+b+c},\;0\leq S_{ij}\leq 1$$

such that

a. The number of items that visit both devices
b. The number of items that visit device i but not device j
c. The number of items that visit device j but not device i

Similarity coefficient can be used to group items based on the level of similarities they share. Several mathematical algorithms are available for defining and forming a unified family associated with the formation of a cell made up of different machines, such as clustering algorithms that utilise the similarity coefficient between the parts to be formed on these machines. The similarity coefficients between machines/parts are computed using formulas of specific similar equations. These similarity coefficients are then organised into a matrix, which is called the similarity matrix. The similarity matrix is then provided as input to the clustering algorithm, for example the single links clustering method (SLINK), to form partial / family cells, where the relevant measure can be the distance or similarity between a pair of items. Groups are obtained by combining nearest neighbours based on their similarity. Results are then displayed graphically in the form of a tree chart, called the dendrogram.

Similarity coefficients between parts or devices are used for creating the dendrogram that represents cells/partial families at different levels of similarity, using the similarity matrix. McAuley used the Jaccard similarity coefficient [38] to address the cell formation problem. The ratio of the number of parts that visit both machines is equal to the number of parts that visit each of the two machines.

The grouping technique is used in conjunction with clustering algorithms to develop groups of suppliers based on the pre-identified similarities between them. An important prerequisite to developing a group of suppliers is the creation of a comprehensive list that contains all factors deemed important by decision-makers, which are critical for the implementations of supply chain management.

The proposed similarity factor (SC) determines the relationship between suppliers with respect to specific criteria developed by decision-makers.

## Triangle fuzzy number (TFN)

Linguistic variables that are designated by decision-makers in the firm and relate to the importance of each supplier with respect to a certain factor can be fuzzy. Word qualifiers, such as “extremely high”, “very high”, “high”, “medium”, “low”, “very low”, and “extremely low” can be used to show subjective attributes related to the factors under consideration. On the other hand, qualifiers such as “extremely good”, “very good”, “good”, “fair”, “poor”, “medium”, “very low”, and “extremely low” can represent conditional attributes.

**Fig. 1:**
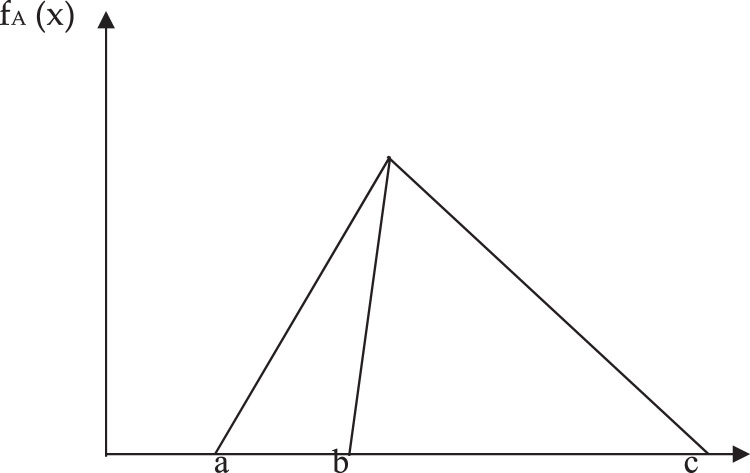
Linguistic variable, membership function.

These linguistic characteristics can be better expressed in terms of trigonometric triangles, which assign a fragile/fuzzy number to a linguistic term by eliminating confusion. The triangle figure can be used to express linguistic variables. Deng [39] discusses this mathematical representation of the TFN. The triangle is shown in Fig. 1, denoted by *A* = (a, b, c), where the letter ``a'' is the minimum and ``c'' is the upper limit. Function f_A_ (x) is called the membership function for the triangle number and is given by the following conditions:

(2) $$f_{A}\left(x\right)=\left\{ \begin{array}{c} \left(x-a\right)/\left(b-a\right);\;if\;\;a\;\leq x\leq \;b\\ \left(c-x\right)/\left(c-b\right);\;if\;\;b\;\leq x\;\leq c\\ 0;\;\;\;\;\;if\;\;a,x>c \end{array}\right.$$

Several conversion measures have been developed to measure the density of positions developed by decision-makers in the form of linguistic terms, such as a variety of Likert scales, where a language variable recognised by a decision-maker can be converted into a fuzzy group using an ambiguous measure in the form of a triangle, as shown in Fig. 1. Here, we used Saaty scale from 0 to 9 to represent the importance of the criteria under consideration that are listed in Table 1, and in each row the three comma-separated numbers correspond to the variables a, b, c, as defined above.

**Table 1:** Conversion of language variables to fuzzy numbers.

Saaty scale	Definition	Fuzzy triangle	
9.0	Absolutely Important	(9, 9, 9)	AI
7.0	Strongly Important	(6, 7, 8)	SI
5.0	Fairly Important	(4, 5, 6)	FI
3.0	Weakly Important	(2, 3, 4)	WI
1.0	Equally Important	(1, 1, 1)	EI

To represent fuzzy data, a linguistic variable is used as an indicator of the data type, when full information about the system under consideration is not provided. Crisp data can be used with a decision matrix. The following matrix describes a multidimensional fuzzy situation with *m* alternatives with *n* criteria.

(3) $$\begin{array}{ccc} & & C_{1}C_{2\ldots .}C_{n}\\ & & D=\begin{array}{c} A_{1}\\ A2\\ Am \end{array}\left|\begin{array}{c} x11\ldots x12\ldots x1n\\ x21\ldots x22\ldots x2n\\ xm1\ldots xm2\ldots xmn \end{array}\right| \end{array}$$

Such that

*x_ij_ = (a_ij_, b_ij,_ c_ij_)* ………………..….….triangle fuzzy numbers (TFNs)

*A* = A_1_, A_2_, ……, A_m_ ……………..……..alternatives and

*C = C_1_,* C_2m_ *_…….,_ C_n_* ……………………..the criteria

## Defuzzification processes

Defuzzification amounts to converting the TFN into a crisp number. Literature survey indicates that many defuzzification processes have been developed in the context of manufacturing research [39]. The gradual mean integration representation [[40], [41], [42]] will be used in the TFN statement, where the representation of the gradual integration of TFN = (*n_1_, n_2_, n_3_*) is equal to

(4) $$P\left(N\right)=\left(n_{1}+4n_{2}+n_{3}\right)/6$$

## Algorithm development

The algorithm used is based on the algorithm of Abdelhadi [43], mainly, a similarity matrix between all suppliers is developed, then grouping the suppliers based on their maximum similarities using clustering algorithms which can be described as follow:

Step 1: Decision makers choose alternatives (suppliers) based on the importance of their needs.
Step 2: Decision makers define evaluation criteria for evaluating suppliers.
Step 3: The appropriate language variables are selected to evaluate the different variables, based on their importance.
Step 4: The total weight of each alternative is determined based on the selection range.
Step 5: A fuzzy decision matrix is generated and transformed into a clear and pure data representation, using Eq. (4).
Step 6: The matrix of similarity coefficients is the ambiguous scaled-weighted matrix because it ranks the alternatives according to the importance of the criteria based on the assessment of decision makers.
Step 7: A dendrogram is generated using a specific aggregation algorithm, which produces groups of suppliers connected according to the level of similarity between them.

## Applying the proposed method to supplier selection

In this section, a hypothetical example is used to illustrate the proposed method using microsfot excel to imlement all calculations. An assumption of a situation in which a company plans to develop a strategy by selecting from different suppliers, A*_i_* and each supplier has its own characteristics (step 1). Selection of suppliers will be based on grouping the suppliers into groups based on five different criteria proposed by the company's decision-makers, who are familiar and working with the different suppliers, and can easliy judge them for each one of the criteria. Hence, we assume that the information provided by each decision maker is valid.

Criteria selected by decision-makers DM*_i_* include the following variables: C1, Reliability; C2, Quality; C3, Product functions as per customer's request; C4, Suppliers just on time delivery and C5, Supplier delivery time (lead time) (step 2). Table 2 lists decision-makers’ judgments about each resource according to pre-determined analysis using linguistic variables (step 3), while Table 3 lists the transformed crisp values obtained from the de-obfuscation (defuzzification) process.

**Table 2:** Decision-makers’ judgements for each supplier.

		DM1	DM2	DM3	DM4
C1	A1	EI	AI	FI	FI
	A2	EI	FI	SI	SI
	A3	EI	SI	FI	AI
	A4	EI	AI	FI	FI
C2	A1	AI	FI	AI	FI
	A2	EI	SI	FI	SI
	A3	FI	AI	SI	SI
	A4	SI	FI	AI	SI
C3	A1	AI	FI	SI	EI
	A2	FI	SI	SI	EI
	A3	SI	FI	EI	FI
	A4	AI	FI	FI	FI
C4	A1	SI	EI	EI	FI
	A2	EI	SI	FI	SI
	A3	SI	WI	SI	AI
	A4	FI	WI	WI	FI
C5	A1	FI	EI	FI	SI
	A2	SI	SI	FI	FI
	A3	WI	FI	SI	WI
	A4	EI	EI	WI	EI

**Table 3:** Lists the transformed crisp values obtained by defuzzification process.

		DM1	DM2	DM3	DM4
C1	A1	(1, 1, 1)	(9, 9, 9)	(4, 5, 6)	(4, 5, 6)
	A2	(1,1,1)	(4,5,6)	(6,7,8)	(6,7,8)
	A3	(1,1,1)	(6,7,8)	(4,5,6)	(9,9,9)
	A4	(1,1,1)	(9,9,9)	(4,5,6)	(4,5,6)
C2	A1	(9, 9, 9)	(4, 5, 6)	(9, 9, 9)	(4, 5, 6)
	A2	(1, 1, 1)	(6, 7, 8)	(4, 5, 6)	(6, 7, 8)
	A3	(4, 5, 6)	(9, 9, 9)	(6, 7, 8)	(6, 7, 8)
	A4	(6, 7, 8)	(4, 5, 6)	(9, 9, 9)	(6, 7, 8)
C3	A1	(9, 9, 9)	(4, 5, 6)	(6, 7, 8)	(1, 1, 1)
	A2	(4, 5, 6)	(6, 7, 8)	(6, 7, 8)	(1, 1, 1)
	A3	(6, 7, 8)	(4, 5, 6)	(1, 1, 1)	(4, 5, 6)
	A4	(9, 9, 9)	(4, 5, 6)	(4, 5, 6)	(4, 5, 6)
C4	A1	(6, 7, 8)	(1, 1, 1)	(1, 1, 1)	(4, 5, 6)
	A2	(1, 1, 1)	(6, 7, 8)	(4, 5, 6)	(6, 7, 8)
	A3	(6, 7, 8)	(2, 3, 4)	(6, 7, 8)	(9, 9, 9)
	A4	(4, 5, 6)	(2, 3, 4)	(2, 3, 4)	(4, 5, 6)
C5	A1	(4, 5, 6)	(1, 1, 1)	(4, 5, 6)	(6, 7, 8)
	A2	(6, 7, 8)	(6, 7, 8)	(4, 5, 6)	(4, 5, 6)
	A3	(2, 3, 4)	(4, 5, 6)	(6, 7, 8)	(2, 3, 4)
	A4	(1, 1, 1)	(1, 1, 1)	(2, 3, 4)	(1, 1, 1)

The TFN numbers *x_ij_ = (a_ij_, b_ij,_ c_ij_)* shown in Table 4 were used as Low, medium and high crisp values after defuzzification. Table 4 consists of three sections, the first, second and third sections have the low (a_ij_), medium (b_ij_), high (c_ij_) crisp values respectively for all alternatives and criteria assigned by different decision makers. Then, in each section, the sum of all crisp values attained from defuzzification was computed for each alternative and criterion from the different decision makers and this sum was used to find the gradual mean integration using Eq. (4), where n_1_, n_2_, n_3_, represents the sum of crisp values (for a particular alternative and criterion) from the different decision makers from the first, second, third part of Table 4, respectively (step 5).

**Table 4:** Crisp values obtained after defuzzification at Low, medium and high values.

low						
		DM1	DM2	DM3	DM4	Total
C1	A1	1	9	4	4	18
	A2	1	4	6	6	17
	A3	1	6	4	9	20
	A4	1	9	4	4	18
C2	A1	9	4	9	4	26
	A2	1	6	4	6	17
	A3	4	9	6	6	25
	A4	6	4	9	6	25
C3	A1	9	4	6	1	20
	A2	4	6	6	1	17
	A3	6	4	1	4	15
	A4	9	4	4	4	21
C4	A1	6	1	1	4	12
	A2	1	6	4	6	17
	A3	6	2	6	9	23
	A4	4	2	2	4	12
C5	A1	4	1	4	6	15
	A2	6	6	4	4	20
	A3	2	4	6	2	14
	A4	1	1	2	1	5
medium						
		DM1	DM2	DM3	DM4	Total

C1	A1	1	9	5	5	20
	A2	1	5	7	7	20
	A3	1	7	5	9	22
	A4	1	9	5	5	20
C2	A1	9	5	9	5	28
	A2	1	7	5	7	20
	A3	5	9	7	7	28
	A4	7	5	9	7	28
C3	A1	9	5	7	1	22
	A2	5	7	7	1	20
	A3	7	5	1	5	18
	A4	9	5	5	5	24
C4	A1	7	1	1	5	14
	A2	1	7	5	7	20
	A3	7	3	7	9	26
	A4	5	3	3	5	16
C5	A1	5	1	5	7	18
	A2	7	7	5	5	24
	A3	3	5	7	3	18
	A4	1	1	3	1	6
high						
		DM1	DM2	DM3	DM4	

C1	A1	1	9	6	6	22
	A2	1	6	8	8	23
	A3	1	8	6	9	24
	A4	1	9	6	6	22
C2	A1	9	6	9	6	30
	A2	1	8	6	8	23
	A3	6	9	8	8	31
	A4	8	6	9	8	31
C3	A1	9	6	8	1	24
	A2	6	8	8	1	23
	A3	8	6	1	6	21
	A4	9	6	6	6	27
C4	A1	8	1	1	6	16
	A2	1	8	6	8	23
	A3	8	4	8	9	29
	A4	6	4	4	6	20
C5	A1	6	1	6	8	21
	A2	8	8	6	6	28
	A3	4	6	8	4	22
	A4	1	1	4	1	7

In the sixth step, the resulted crisp gradual means integral TFN values listed in Table 5 provides a unifrom load factor to each supplier depending on the criteria chosen by the decision makers, these weights. are shown by the decision matrix shown in Table 6, which are used to build the dendrogram. The dendrogram is constructed using clustering algorithms, such as single clustering or complete clustering algorithms. The dendrogram reveals groups/clusters of suppliers, which reflect their similarities.

**Table 5:** The crisp gradual means integral TFN.

Criteria	Supplier	The gradual mean integration
C1	A1	20
	A2	20
	A3	22
	A4	20
C2	A1	28
	A2	20
	A3	28
	A4	28
C3	A1	22
	A2	20
	A3	18
	A4	24
C4	A1	14
	A2	20
	A3	26
	A4	16
C5	A1	18
	A2	24
	A3	18
	A4	6

**Table 6:** The weight per criterion assigned by the decision-makers for each supplier.

Criteria						
		C1	C2	C3	C4	C5
Supplier	A1	20	28	22	14	18
	A2	20	20	20	20	24
	A3	22	28	18	26	18
	A4	20	28	24	16	6

In the last step (step 8), we use commercially available software, Minitab, which employs a mathematical algorithm to build clusters. An appropriate clustering algorithm, such as single linkage grouping/clustering or complete linkage clustering, can be selected. Complete linkage clustering is used for constructing the dendrogram, which will be considered and examined to reveal similarities between suppliers as they relate to the standards set by the decision-makers.

**Fig. 2:**
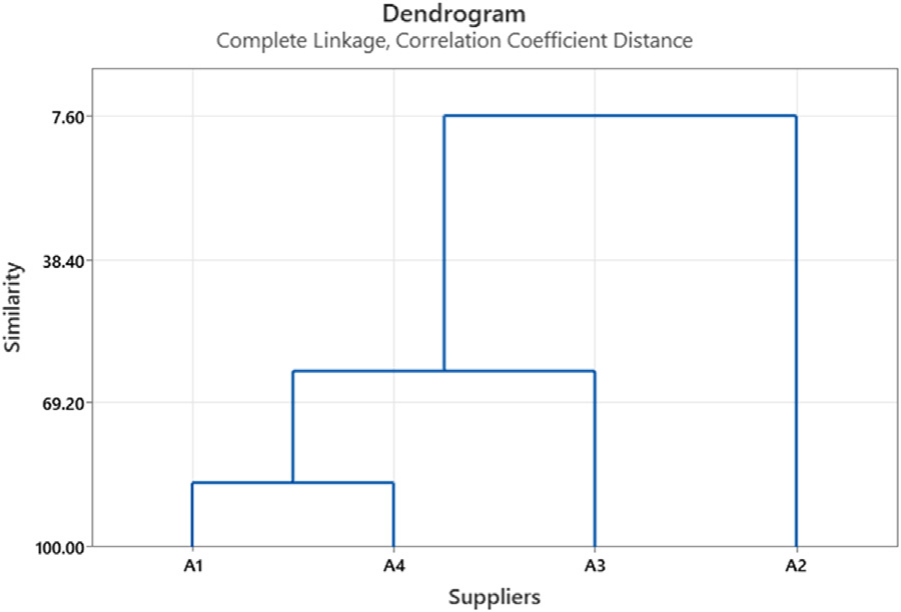
Clusters of Suppliers at different levels of similarity.

**Fig. 3:**
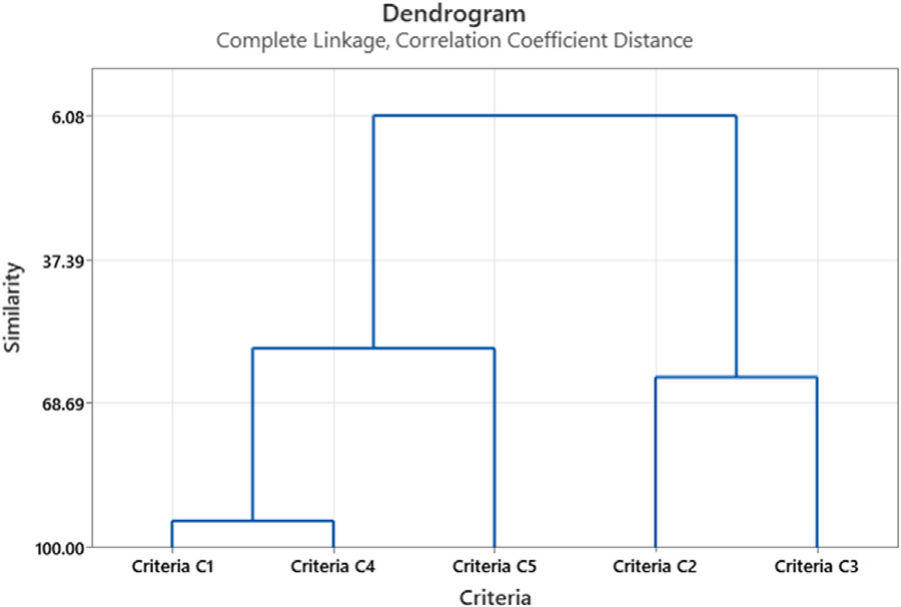
Clusters of Criteria at different levels of similarity.

Several insights can be obtained using this method, such as clustering suppliers based on their similarity, hence the best supplier (priority) that the company must select which is a typical decision that a supplier selection method usually provides; for example, management can construct a dendrogram that consists of one cluster only, and study it, to see how suppliers are joined together based on the similarities between them. Fig. 2 shows how all suppliers are joined together, at different levels of similarity. It is clear that the similarity measure for suppliers A1 and A4 at approximately 86 % while the similarity between suppliers A2 and A3 is at about 62 %. Hence, the decision maker can use this similarity level to choose between suppliers based on the same preferences and the similarity level between the suppliers, for example, the decision makers can substitute A1 by A4 or vice versa since the level of similarities between them is very high based on the initial decision making. However, neither A1 nor A4 can be a substitute for either A2 and/or A3 since their level of similarity is only at 7 %. Likewise, referring to the dendrogram shown in Fig. 3, it is clear that C2 and C4 are at high level of similarity (approximately about 90 %), which indicate that either of them can substitute the other. The same procedure can be applied to the rest of the suppliers in this problem. Note that the dendrogram reveals that criteria number C5 is apparently an outlier (Table 7).

**Table 7:** Matrix showing 9 suppliers with 5 criteria.

	C1	C2	C3	C4	C5
A1	14.2	23.8	18	23.8	21.8
A2	18	18	24	20	14.2
A3	21.8	12.2	21.8	16.2	20
A4	16.2	25.7	21.8	22	23.8
A5	20.1	20.9	21	23	23.8
A6	20.2	22.4	20.1	22	20.9
A7	21.4	23	20.4	21.1	20.8
A8	22	23	20.9	22	23
A9	23	22	23.8	23	22

**Fig. 4:**
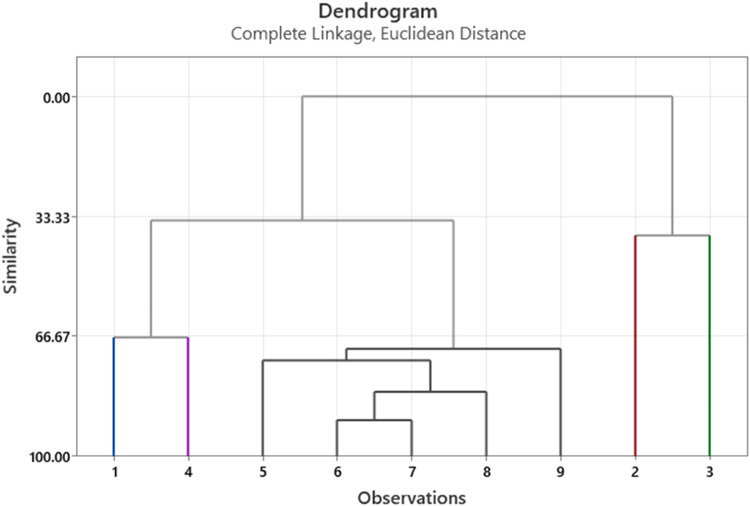
Dendrogram with 9 suppliers.

To validate the results from another aspect, we used the same approach on a selected article called, Preventive Maintenance Operations Scheduling Based on Eigenvalue and Clustering Methods [43]. Authors in [43] used clustering algorithm to generate groups based on eigenvalue developed based on the similarity using failure–machine incidence matrix. The results using the approach used in this research is comparable to the result found in the mentioned article. It is worth mentioning that the presented example consists of 4 suppliers, which may not give a practical view about the real-life situation. However, it was presented in its simple form to show the proposed methodology and to save space in writing this article. If a hypothetical number of suppliers is reached 9 as shown in table, then the number of tables provided to explain this approach will be more than doubled (more than 16 tables) which will make it difficult for the reader to follow. It can be seen that if we have 9 suppliers as shown in 7, then the dendrogram will be shown in Fig. 4, it is clear that suppliers 6 and 7 are having the highest similar-ty at 89.9 % as seen in Table 8, which presents the formation of the clusters based on their similarity using single linkage clustering.

**Table 8:** Similarity level of grouping suppliers.

Step	Number of clusters	Similarity level	Distance level	Clusters joined		New cluster
1	8	89.93	1.64	**6**	**7**	6
2	7	82.02	2.94	**6**	**8**	6
3	6	73.31	4.36	**5**	**6**	5
4	5	70.12	4.88	**5**	**9**	5
5	4	66.92	5.41	**1**	**4**	1
6	3	38.58	10.04	**2**	**3**	2
7	2	34.46	10.72	**1**	**5**	1
8	1	0.0000	16.36	**1**	**2**	1

Hence, this proposed approach can make use of suppliers based on their similarity and the need of the company to select the best supplier from the designated group.

## Discussion and conclusion

This research addressed the issue of grouping different suppliers in clusters based on the similarities between them according to the criteria set by decision-makers. The current disruption in the supply chain management due to the political issues and wars leads to changes in the business plans which include supply chain management's plan revision. This research addresses the issue of changing the suppliers based on the dynamic of the selection of suppliers. This research distinguish itself from other researches mainly by converting the fuzzy decision makers’ selection of alternatives (suppliers) based on the importance of their needs into an appropriate language variable. Then a fuzzy decision matrix is generated and transformed into a clear and pure data representation.

In this work, an attempt to join suppliers based on the highest similarities between them, based on the objectives of the decision-makers, which in turn eases the supply-chain processes and alleviates the above-mentioned problems. This paper attempts to explore priority coordination mechanisms and prioritisation of selected partners using group technology methods based on using Crisp Gradual Means Integral which is a method being used as conversion measures to measure the density of positions developed by decision-makers in the form of linguistic terms. This research will response positively to the changes of the variability in the risks of the of each entity and deals with coordination of the supply chain process dynamically. Using the proposed approach will lead to minimize the risk of meeting the goals of the manufacturing firms. By grouping suppliers into clusters, several advantages can be gained by firms. For example, a firm can determine the level of similarity between different suppliers when taking into consideration all variables of interest to its decision-makers. When computing this similarity, the decision-makers can substitute any of the suppliers with any other from the same group in the event of the occurrence of a negative event that prevents the selected supplier from fulfilling his/her obligations. For example, referring to Table 8, suppose that supplier 8 was unable to deliver the results; then, supplier 6 can serve as a replacement, with the expected outcome at 82 %. This approach provides decision-makers an ability to design their own groups of suppliers, or they can choose the number of groups they can divide their suppliers into, which will allow them to make a correct decision according to their own circumstances. In addition, decision-makers can select their group of suppliers based on the criteria they developed. This research can be expanded to incorporate geographical location or any other factors pertaining to suppliers and of relevance to decision-makers.

## Declaration of Competing Interest

The authors declare that they have no known competing financial interests or personal relationships that could have appeared to influence the work reported in this paper.

## Data Availability

No data was used for the research described in the article.
